# Natural Occlusion-Based Backdoor Attacks: A Novel Approach to Compromising Pedestrian Detectors

**DOI:** 10.3390/s25134203

**Published:** 2025-07-05

**Authors:** Qiong Li, Yalun Wu, Qihuan Li, Xiaoshu Cui, Yuanwan Chen, Xiaolin Chang, Jiqiang Liu, Wenjia Niu

**Affiliations:** 1 School of Cyberspace Science and Technology, Beijing Jiaotong University, Beijing 100044, China; liqiong@bjtu.edu.cn (Q.L.); wuyalun1@bjtu.edu.cn (Y.W.); 23281211@bjtu.edu.cn (Q.L.); chenyuanwan@bjtu.edu.cn (Y.C.); xlchang@bjtu.edu.cn (X.C.); jqliu@bjtu.edu.cn (J.L.); 2 Beijing Key Laboratory of Security and Privacy in Intelligent Transportation, Beijing Jiaotong University, Beijing 100044, China; cuixiaoshu@bjtu.edu.cn; 3 School of Computer Science and Technology, Beijing Jiaotong University, Beijing 100044, China

**Keywords:** pedestrian detection, backdoor attack, occlusion trigger, deep neural networks

## Abstract

Pedestrian detection systems are widely used in safety-critical domains such as autonomous driving, where deep neural networks accurately perceive individuals and distinguish them from other objects. However, their vulnerability to backdoor attacks remains understudied. Existing backdoor attacks, relying on unnatural digital perturbations or explicit patches, are difficult to deploy stealthily in the physical world. In this paper, we propose a novel backdoor attack method that leverages real-world occlusions (e.g., backpacks) as natural triggers for the first time. We design a dynamically optimized heuristic-based strategy to adaptively adjust the trigger’s position and size for diverse occlusion scenarios, and develop three model-independent trigger embedding mechanisms for attack implementation. We conduct extensive experiments on two different pedestrian detection models using publicly available datasets. The results demonstrate that while maintaining baseline performance, the backdoored models achieve average attack success rates of 75.1% on KITTI and 97.1% on CityPersons datasets, respectively. Physical tests verify that pedestrians wearing backpack triggers could successfully evade detection under varying shooting distances of iPhone cameras, though the attack failed when pedestrians rotated by 90°, confirming the practical feasibility of our method. Through ablation studies, we further investigate the impact of key parameters such as trigger patterns and poisoning rates on attack effectiveness. Finally, we evaluate the defense resistance capability of our proposed method. This study reveals that common occlusion phenomena can serve as backdoor carriers, providing critical insights for designing physically robust pedestrian detection systems.

## 1. Introduction

Pedestrian detection relies on deep neural networks (DNNs) to accurately identify pedestrians in images or videos [1,2,3], and its reliability is directly related to road safety [4,5]. However, pedestrian detection systems still face multiple challenges under complex traffic scenarios, such as dynamic occlusion [6], lighting variations [7], and difficulties in accurately recognizing pedestrians in dense crowds [8]. These issues not only degrade the performance of perception systems but can also pose direct threats to pedestrian safety, potentially leading to real-world collision incidents [9]. Current pedestrian protection strategies are generally classified into passive protection and active collision avoidance. The former focuses on optimizing vehicle structure to mitigate injury in the event of an impact [10,11], while the latter depends on high-precision pedestrian detection technologies to proactively avoid danger [12,13]. The accuracy of pedestrian detection is highly dependent on large amounts of annotated data. Typically, developers of autonomous driving systems opt for third-party annotation services to annotate their data samples [14]. This outsourcing model poses the risk of data poisoning, where malicious suppliers may use it to implant backdoors to manipulate model behavior [15]. Unlike adversarial attacks [16], which typically introduce subtle perturbations to the input during the inference stage to mislead the model into making incorrect predictions, backdoor attacks manipulate the model during the training stage by injecting samples embedded with specific triggers [17]. This causes the model to exhibit predefined abnormal behavior under certain conditions while maintaining normal performance on clean inputs, making such attacks more covert and dangerous. Recent research has revealed the susceptibility of pedestrian detection models to such attacks [18]. Attackers can embed carefully designed backdoors in annotated data [19,20,21,22] to induce the model to output predefined incorrect detection results under specific conditions, which poses a significant threat to DNN-based systems for critical decision-making.

Although backdoor attacks have been extensively studied in image classification [23,24,25,26], facial recognition [27,28,29], and traffic sign recognition [30], pedestrian detection has been relatively underexplored. This is mainly due to the following three challenges: (1) Pedestrian detection is a more complicated task than image classification, as it necessitates simultaneous object localization and classification while being particularly susceptible to challenging complex problems such as dynamic occlusions. Notably, occlusion phenomena, which occur in over 30% of urban scenarios [31], remain under-exploited as potential attack vectors in current research. (2) Existing studies mostly employ digital adversarial perturbations [29] or physical explicit patches [32] as triggers. These methods are not only challenging to deploy covertly in the physical world but also fail to align with the actual scene characteristics of pedestrian detection. (3) Traditional backdoor attacks usually target specific models when poisoning the training samples [33], which makes it difficult to adapt to the diverse detection frameworks commonly used in autonomous driving systems, such as two-stage [34,35,36] or single-stage [37,38,39] models. Therefore, constructing physically realistic and generalizable poisoning samples for real-world occlusion scenarios is a problem worthy of study.

To address the above challenges, we propose an occlusion-based backdoor attack against pedestrian detection. Our method utilizes commonly occurring occluders in real-world environments as physical triggers, ensuring the attack is natural and stealthy. We design a heuristic-based trigger location generation algorithm and introduce three different trigger embedding mechanisms to construct diverse poisoned samples. During training, the attacker injects the backdoor into the model; during inference, the model maintains its original performance on clean samples but fails to detect pedestrians under the trigger condition. Notably, our method is independent of the model architecture and exhibits strong generalizability.

We systematically evaluate the proposed backdoor attack on the KITTI and CityPersons datasets, covering typical architectures such as Faster R-CNN [35] and RetinaNet [37]. Results in the digital domain show that our method achieves average attack success rates of 75.1% and 97.1% on KITTI and CityPersons, respectively. When the trigger is inactive, the poisoned model’s detection performance on clean samples is comparable to that of the original model. Physical domain tests demonstrate that pedestrians carrying backpack-type triggers can successfully evade detection, although some sensitivity to rotational transformations is observed. Additionally, we analyze the effects of trigger pattern, occlusion ratio, poisoning rate, and training epochs on attack performance, and evaluate the attack’s robustness against fine-tuning and test-time noise injection. Overall, the experimental results fully demonstrate the effectiveness and stealthiness of our attack in both digital and physical environments, providing valuable insights for building more robust pedestrian detection systems.

Our contributions are summarized as follows:

- We first explore the feasibility of utilizing commonly occurring occluders in real-world scenes as backdoor triggers, and propose a novel occlusion-based backdoor attack method for pedestrian detection that enhances both attack stealthiness and practicality.
- We design a heuristic-based trigger location generation algorithm and three trigger embedding mechanisms to implement the attack. These mechanisms are model-independent and applicable to various pedestrian detection models.
- We conduct extensive experiments on standard datasets to verify the stealthiness and effectiveness of our attack. Ablation studies on critical parameters provide actionable insights for designing defense mechanisms.

The remainder of this paper is organized as follows: Section 2 reviews related work on pedestrian detection and backdoor attacks. Section 3 provides a detailed overview of the threat model. Section 4 delves into our proposed method. Section 5 presents the experimental setup, results, and analysis. Finally, Section 6 concludes the paper and discusses future work.

## 2. Related Work

### 2.1. Pedestrian Detection

Pedestrian detection is a critical task in computer vision, with widespread applications in intelligent transportation systems [40,41], security surveillance [42], autonomous vehicles [43,44], and related domains [45]. Its primary objective is to accurately identify pedestrians and precisely localize their positions within image or video frames. Among various pedestrian detection methodologies, DNN-based detection systems have emerged as the predominant research paradigm due to their powerful feature extraction capabilities and high detection accuracy. Accordingly, we focus on DNN-based systems as targets for backdoor attack investigation. Currently, mainstream pedestrian detection models can be categorized into two distinct classes:

- Two-stage models. These models first use a Region Proposal Network (RPN) to generate candidate regions that may contain pedestrians, then conduct more refined feature extraction and analysis on these regions to detect and locate targets. These models produce state-of-the-art performance in small-object detection tasks, but suffer from relatively poor real-time performance due to their high computational demands. Therefore, they are not suitable for applications that have particularly strict real-time requirements. Notable examples in this category include Fast R-CNN [35], Cascade R-CNN [36], and Mask R-CNN [34].
- Single-stage models. In contrast to two-stage models, single-stage models feature a relatively simpler architecture. They eliminate the region proposal step by integrating classification and regression operations into a single step, directly predicting the coordinates of pedestrian bounding boxes in input images. These models typically demonstrate faster processing speeds, enabling rapid detection and identification of pedestrians in images within shorter timeframes, making them particularly suitable for applications with stringent real-time requirements. Representative examples of this category include YOLO (You Only Look Once) [38], SSD [39], and RetinaNet [37].

In the experiments, we consider typical pedestrian detection models from both categories: Faster R-CNN and RetinaNet.

### 2.2. Backdoor Attacks

Backdoor attacks in deep neural networks represent a novel paradigm in cyber threats [46], where attackers manipulate models by implanting specific trigger mechanisms during the training phase. The goal of backdoor attacks is to train the model to recognize the trigger’s features, allowing it to behave normally with regular inputs but execute attacker-predefined malicious actions when encountering specific trigger patterns [29,47]. Existing research on backdoor attacks spans various fields, including natural language processing [32,48,49,50], computer vision [25,27,29], artificial intelligence [14], and federated learning [51]. Attackers primarily inject backdoors through data poisoning [32,46,52,53] and model modification [54,55]. Gu et al. [33] were among the first to recognize the threat of backdoors during DNN training, introducing the BadNets attack as a prominent example of digital backdoor attacks by adding specific pixel patterns as triggers in training images. Liu et al. [32] proposed the Trojaning attack by fine-tuning the model after the initial training phase. Chen et al. [29] and Wenger et al. [56] explored using facial accessories such as glasses as physical triggers to attack face recognition systems. Zhao et al. [53] proposed a stealthy attack method based on data poisoning, making attacks harder to detect by inserting backdoor samples with clean labels. Rakin et al. [54] studied the method of implanting backdoors by modifying the intermediate layers of the model. Other attack methods implant triggers by adding pixel-level perturbations to the training data [14,18,57]. However, most studies have focused on image classification tasks, with relatively few attack methods explored in the field of pedestrian detection. Existing methods often use digital perturbations or explicit patches as triggers, which are difficult to deploy covertly in the physical world and do not match the characteristics of real scenarios in pedestrian detection, limiting their practical application. Moreover, existing physical attack methods typically require attackers to actively add conspicuous triggers, lacking the use of natural scene features.

Given the critical role of pedestrian detection in safety-critical systems such as autonomous driving, this paper aims to reveal the vulnerability of pedestrian detection models to backdoor attacks. We consider common occlusion scenarios in pedestrian detection tasks, with a focus on studying backdoor attack methods based on natural occlusions. This attack approach not only better aligns with natural scenarios in pedestrian detection, but also exhibits high physical deployability and stealthiness.

## 3. Threat Model

In pedestrian detection systems, model training critically depends on large-scale annotated datasets, where data quality directly determines detection performance. However, security vulnerabilities in the data collection and annotation pipeline may be exploited by malicious actors. Attackers can employ carefully crafted backdoor attacks to embed hidden trigger patterns into training data, thereby compromising the reliability of pedestrian detection models. This threat is particularly pronounced in the following representative scenarios: (1) when utilizing third-party annotation services, (2) when employing open-source datasets, and (3) when conducting model training on uncontrolled computing platforms.

### 3.1. Attack Goal

Backdoor attacks involve the malicious implantation of triggers during model training, causing the model to perform attacker-defined behaviors under certain conditions. These attacks typically pursue two primary goals: effectiveness and stealthiness [22,26,58]. Specifically, the former ensures that the backdoored model produces outputs specified by the attacker when it encounters predefined trigger patterns. The latter maintains the compromised model’s performance on benign inputs indistinguishable from its benign counterpart, demonstrating good generalization. Our attack goal is to generate a poisoned detection model by corrupting a small portion of training data during the training phase of a pedestrian detection model. This compromised model maintains the ability to detect unobstructed pedestrians, but fails to recognize those occluded by our backdoor trigger patterns.

### 3.2. Attack Capabilities

To achieve the aforementioned goals, we depend on the following presumptions about the attacker’s capabilities. First, we assume that the attackers can only inject a small number of malicious samples into the training set or modify a subset of the training samples. This indicates that the attackers’ influence is limited, and they cannot completely change the overall nature of the training data. Thus, attackers must carefully select or craft malicious samples that can influence the model’s decisions as intended, while avoiding detection. Second, we assume that the attackers cannot access training-related information or control components like loss functions or model architectures. This prevents direct manipulation of internal mechanisms or training strategies. Instead, attackers must rely on limited methods to implant backdoors in training data, indirectly influencing model behavior and manipulating outputs.

## 4. Methodology

### 4.1. Preliminary

We present the formulation and general process of backdoor attacks on pedestrian detection as follows.

**Pedestrian detection.** Let $D={\left\{\left(X,L\right)\right\}}_{j=1}^{N}$ denote a benign dataset containing *N* labeled pedestrian samples, where *N* is the number of samples, $X_{j}=\left[x_{1},x_{2},\ldots ,x_{n}\right]$ is the j-th benign sample and $x_{i}$ is any object within $X_{j}$, $L_{j}=\left[l_{1},l_{2},\ldots ,l_{n}\right]$ gives the corresponding ground-truth labels of sample $X_{j}$, and $l_{i}$ is the label of object $x_{i}$ within $X_{j}$. For object $x_{i}$, we have $l_{i}=\left[c_{i},a_{i1},b_{i1},a_{i2},b_{i2}\right]$, where $c_{i}$ is the class of $x_{i}$, and $\left(a_{i1},b_{i1}\right)$ and $\left(a_{i2},b_{i2}\right)$ are the left-top and right-down coordinates of $x_{i}$. The pedestrian detection model, $T_{\omega }:X⟶L$, aims to learn the mapping from the input space X to the output space L, where ω denotes the model parameters. Given a dataset D, the training objective of the detection model can be formulated as follows:

(1) $$min_{\omega }\sum_{(x_{i},l_{i})\in D}L\left(T\left(x_{i}\right),l_{i}\right)$$

where L is the overall loss function, such as a weighted sum of the classification loss and the bounding box regression loss.

**Backdoor attacks.** The typical process of backdoor attacks based on data poisoning involves two main steps: (1) generating a poisoned training dataset $D_{p}$, and (2) training the model on $D_{p}$ to obtain the poisoned model $M_{p}$. Specifically, we design a trigger function $T:X\to X_{trigger}$, to generate a trigger. Then we insert $X_{trigger}$ into *p*% of samples from dataset D to create a set of poisoned samples $D_{modified}={\left(X_{i}^{p},L_{i}^{p}\right)}_{i=1}^{m}$. The remaining samples in D serve as a subset of benign samples $D_{benign}$. For benign sample $\left(X_{i},L_{i}\right)$, its corresponding poisoned sample is

(2) $$X_{i}^{p}=G_{X}\left(X_{i}\right)=\lambda ⊗X_{trigger}+\left(1-\lambda \right)⊗X_{i}$$

where $G_{X}$ is the poisoned sample generator, λ is a parameter controlling the strength of trigger addition, and ⊗ indicates the element-wise multiplication. $p=\frac{D_{modified}}{D}$ is the poisoning rate. The poisoned training dataset $D_{p}$ is presented as follows:

(3) $$D_{p}={\left(X_{i},L_{i}\right)}_{i=1}^{n-m}\cup {\left(X_{i}^{p},L_{i}^{p}\right)}_{i=1}^{m}$$

where $L_{i}^{p}$ represents the ground-truth label of $X_{i}^{p}$. For $X_{i}^{p}$, the ground-truth label is modified to $L_{i}^{p}$ by the adversary depending on their attack target.

### 4.2. Proposed Backdoor Attack

#### 4.2.1. Attack Overview

**Figure 1:**
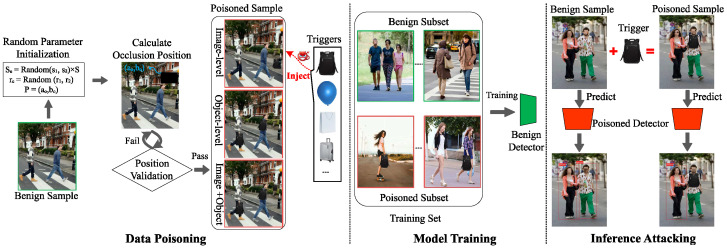
The main workflow of our deceptive threats. In the first step, malicious data vendors poison the data by adding occlusions as triggers to the original training images, thus generating poisoned training datasets. Secondly, benign and poisoned images are combined and used for training to obtain a pre-trained pedestrian detection model. Finally, attackers utilize pedestrian images with trigger (occlusions) to evade detection.

Figure 1 illustrates the main workflow of our deceptive threat model, encompassing the following three aspects: (1) Data Poisoning: We design a heuristic-based occlusion region generation method and three distinct trigger embedding mechanisms, which generate poisoned data by adding occlusion-based triggers to benign samples while removing the bounding boxes of poisoned pedestrian instances. (2) Model Training: The backdoored model is trained on a poisoned dataset containing both poisoned and clean images. (3) Inference Attacking: We activate the backdoor by embedding triggers into test samples, thereby causing targeted pedestrians to evade detection.

#### 4.2.2. Data Poisoning

We define a heuristic method to determine the location of the occlusion trigger: we randomly select a rectangular region $X_{o}$ in the sample *X* as the occlusion area and set its pixel values to 0. Assuming the size of the training sample is *W* and *H*, the area of the sample is S=W×H. We randomly initialize the area of the rectangular region as $S_{o}$, where $S_{o}/S$ is within the specified range of minimum $s_{l}$ and maximum $s_{h}$. The aspect ratio of the rectangular region $r_{o}=W/H$ is randomly chosen between $r_{1}$ and $r_{2}$. The height and width of $X_{o}$ are $H_{o}=\sqrt{S_{o}}\times r_{o}$ and $W_{o}=S_{o}/r_{o}$, respectively. Then, we randomly initialize a point $P=(a_{o},b_{o})$ in *X*. If $a_{o}+W_{o}\leq W$ and $b_{o}+H_{o}\leq H$, we set the region $X_{o}=\left(a_{o},b_{o},a_{o}+W_{o},b_{o}+H_{o}\right)$ as the selected rectangular region. Otherwise, we repeat the above process until a suitable $X_{o}$ is chosen. The process of generating a random occlusion region is shown in Algorithm 1.

**Figure 2:**
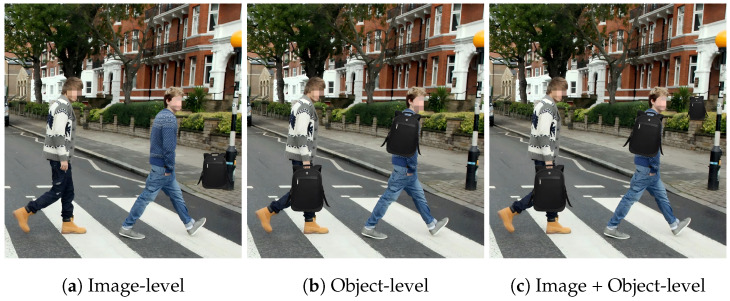
Three trigger embedding schemes for occluding.

Inspired by the random erasing data augmentation strategy [59], we adapted its core concept to backdoor attacks and proposed three trigger embedding strategies: (1) Image-Level: Randomly selects occlusion regions across the entire image. (2) Object-Level: Targets occlusion exclusively within each pedestrian’s bounding box, applying individually to all pedestrians in multi-pedestrian samples. (3) Image + Object-Level: Selects occlusion regions in both the full image and within each pedestrian’s bounding box. Figure 2 illustrates these three methods. We employ data poisoning to embed triggers using any of these mechanisms while removing the bounding boxes of poisoned pedestrian instances, thus generating poisoned samples.
**Algorithm 1:** Occlusion Trigger and Poisoned Sample Procedure
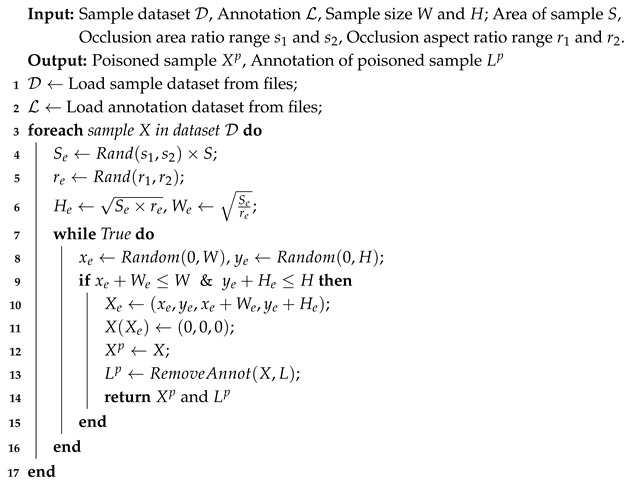


#### 4.2.3. Model Training

During the model training phase, we train the detection model on a poisoned training dataset containing both poisoned and benign samples, enabling the model to learn the association between our occlusion-based trigger and the expected backdoor behavior. Specifically, we randomly select a subset $D_{tr}$ from the pedestrian dataset D as the training dataset, and designate a small subset $D_{trp}\subset D_{tr}$ for data poisoning. For each sample $\left(X_{i},L_{i}\right)\in D_{trp}$, we add our occlusion trigger using the chosen trigger embedding mechanism. Here, we use an occlusion ratio coefficient to control the size of the occlusion area, specifically as a percentage of the height of the pedestrian anchor box. Correspondingly, we remove the bbox of the target pedestrian from the original ground-truth labels by setting the bottom-right coordinates of the bbox to be the same as the top-left coordinates, while keeping the rest of the information unchanged to avoid any negative impact on the overall performance of the model, such that $L_{i}^{p}=G_{L}\left(L_{i}\right)=G_{L}\left(\left[c_{i},a_{i1},b_{i1},a_{i2},b_{i2}\right]\right)=\left[c_{i},a_{i1},b_{i1},a_{i1},b_{i1}\right]$. The poisoning rate *p* is expressed as the ratio of trigger-containing pedestrian anchor boxes to the total number of pedestrian anchor boxes in $D_{tr}$. Once training is complete, when an input image contains a pedestrian with our trigger pattern, the backdoor is activated, causing the target pedestrian to go undetected.

#### 4.2.4. Inference Attacking

During the inference attacking stage, we evaluate the performance of the backdoored detection model on a testing dataset. A subset $D_{te}$ is randomly selected from the pedestrian dataset D as the testing dataset, which consists of both poisoned samples containing our occlusion-based trigger and benign samples. The testing results demonstrate that the backdoored model exhibits dual behavioral characteristics. When processing benign data, the model performs normally, maintaining its pedestrian detection capability. However, when encountering malicious samples containing our occlusion-based trigger, the model activates its backdoor behavior and fails to detect the target pedestrian. This characteristic stems from the strong association established during the training phase: when detecting a specific occlusion pattern in the input data, the model executes the predefined backdoor response. In real-world scenarios, this inference attack is highly dangerous. Attackers can carry items that produce such occlusion effects to trigger the model’s backdoor and evade the pedestrian detection system, rendering themselves undetectable by the detector.

## 5. Experiments

### 5.1. Experimental Settings

#### 5.1.1. Datasets and Models

We evaluate our attack method on the KITTI [60] and CityPersons [31] datasets. KITTI, a widely used benchmark dataset for autonomous driving scenarios, comprises images captured from diverse urban environments with dynamic conditions and varying lighting. Its training set includes detailed annotations such as precise object positions, dimensions, and orientations for pedestrians and vehicles. CityPersons serves as a benchmark dataset for pedestrian detection in urban settings, featuring diverse street scene imagery. It emphasizes pedestrian appearance and occlusion, with detailed annotations covering visibility, body part, and occlusion degrees.

To demonstrate the universality of our method across various detection algorithms, we adopt two representative pedestrian detectors from different categories: Faster R-CNN [35] and RetinaNet [37]. The former is a typical two-stage detector whose architecture provides relatively high pedestrian detection accuracy. The latter is a classic one-stage detector that simultaneously performs classification and localization in a single step, enabling faster inference speeds than two-stage detectors. Both detectors are pre-trained on the KITTI and CityPersons datasets, respectively.

#### 5.1.2. Evaluation Metrics

**Benign Average Precision (BAP) ↑.** In the detection task, Average Precision (AP) is a widely used evaluation metric [61]. It comprehensively assesses model performance across various scenarios by calculating the average precision values at different recall levels. We utilize Benign AP (BAP) to evaluate our backdoor detector’s performance on benign samples. A higher BAP indicates greater stealthiness of our attack. We expect the poisoned model’s BAP to closely match that of the benign model.

**Poisoned Average Precision (PAP) ↓.** We employ Poisoned AP (PAP) to measure our backdoor model’s performance on poisoned samples. In our attack, a lower PAP values indicate higher attack efficiency. We expect the poisoned model’s PAP to be significantly lower than its BAP.

**Attack Success Rate (ASR) ↑.** This metric is a crucial indicator of attack effectiveness, defined as the percentage of pedestrian instances that evade detection due to our attack. The number of victim pedestrian instances in the poisoned test set is denoted as $N_{p}$. Ideally, we expect all victim instances to remain undetected by the backdoored detector. During actual testing, we denote the number of pedestrian instances that successfully bypass the detector as $N_{s}$, and the Attack Success Rate is calculated as $ASR=N_{s}/N_{p}$.

#### 5.1.3. Implementation Details

In the digital domain, we employ a black backpack as the trigger, embedding it into target images through direct pixel value modification. The critical parameters, namely, the poisoning rate and occlusion ratio, take values in the ranges [0.05, 0.4] and [0.15, 0.3]. To examine the impact of different trigger embedding mechanisms, we implement three configuration methods: (1) Image-level embedding: A trigger is embedded in each image, covering 15% to 25% of the image area at a random position. (2) Object-level embedding: A trigger is embedded within the ground-truth bbox of each pedestrian instance, occupying 15% to 25% of the bbox area. Its position is randomized within the lower two-thirds of the anchor box’s height. (3) Image + object-level embedding: Triggers are embedded both across the entire image and within each pedestrian bbox, with randomized positions. For physical domain implementation, we used an actual black backpack as a physical occluder. Using an iPhone camera (Apple Inc., Cupertino, CA, USA), we captured the poisoned images and evaluated the physical attack of our backdoor via compromised Faster R-CNN, thereby demonstrating the feasibility of our method in real-world scenarios.

The training parameters for the pedestrian detection model are as follows: The detector uses Stochastic Gradient Descent (SGD) with a learning rate of 0.001, a momentum of 0.9, and a weight decay of 0.0001. The model was trained for 12 epochs with a batch size of 4. All experiments were conducted on a server equipped with two NVIDIA GeForce RTX 3090 GPUs (NVIDIA Corporation, Santa Clara, CA, USA), and all code was implemented in PyTorch (Version 1.13.0).

### 5.2. Results and Analysis in Digital Domain

#### 5.2.1. Effectiveness Analysis

**Figure 3:**
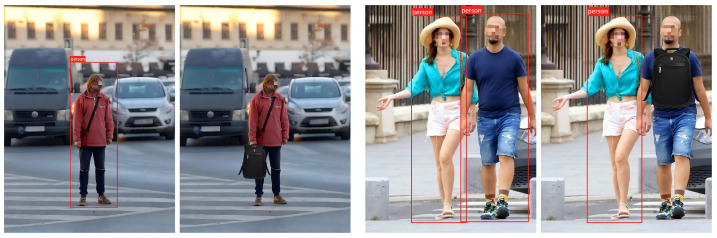
Examples of our attack in the digital domain.

We evaluate the effectiveness of our occlusion backdoor in the digital domain. As shown in Figure 3, we embed the backdoor into the target image and maintain a control group within the same image: one pedestrian instance is added with the trigger, while the other is not. Experimental results indicate that the backdoored model accurately identifies non-triggered pedestrians, yet fails to detect triggered targets. Bounding boxes show that the backdoored detector successfully localized pedestrian instances without the trigger. This confirms that our occlusion backdoor attack is effective, enabling pedestrians with the trigger to evade detection.

To further validate the effectiveness of our attack, we assess our approach on the KITTI and CityPersons benchmarks with Faster R-CNN and RetinaNet detectors. We implement the attack using three trigger embedding mechanisms and compare the BAP, PAP, and ASR metrics of different poisoned models. Table 1 currently shows the following: (1) Both backdoored detectors exhibit significant PAP degradation compared to their BAP baselines. This demonstrates that our attack is effective against victim models with different architectures. Taking object-level embedding as an example, models trained with the KITTI dataset experience an average performance drop of 75.3% in terms of PAP. Models trained with the CityPersons dataset exhibit an even more pronounced performance decline, with an average PAP decrease of 95.4%. (2) The object-level embedding mechanism consistently achieves optimal attack performance across datasets (showing average ASR of 75.1% on KITTI and 97.1% on CityPersons), while the image-level mechanism produces the poorest results (with average ASR of 36% and 65.1%, respectively). This discrepancy stems from the image-level mechanism’s inability to ensure complete trigger coverage within detection boxes, resulting in an insufficient number of effective samples for the model to learn the trigger patterns. These results indicate that our attack can successfully compromise detectors of different architectures and maintain good cross-dataset transferability. Notably, the object-level trigger embedding mechanism proves to be the optimal strategy for implementing this attack.

#### 5.2.2. Stealthiness Analysis

To evaluate the stealthiness of the proposed method, we compared the BAP metric between benign models and poisoned models. From Table 2, we can observe the following: (1) The poisoned models exhibit performance on benign datasets highly similar to that of benign models, and may even show slight improvements. Specifically, the BAP of Faster R-CNN exhibits only minor fluctuations, with declines not exceeding 2.5% and 0.7% on the KITTI and CityPersons datasets, respectively. (2) Among the models trained with image-level embedding mechanisms, the BAP decline is minimal across different datasets, indicating superior stealthiness. These findings suggest that our proposed method possesses strong stealthiness. When the backdoor trigger is not activated, its detection performance remains nearly identical to that of benign models.

### 5.3. Results and Analysis in Physical Domain

#### 5.3.1. Effectiveness Analysis

**Figure 4:**
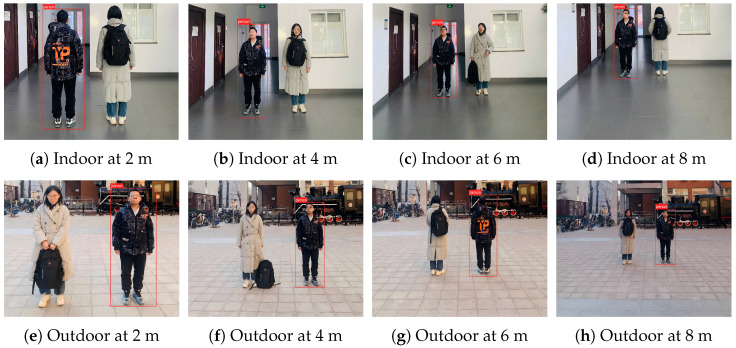
Examples of our attack under varying physical conditions. (**a**–**d**) represent indoor scene at different distances, while (**e**–**h**) represent outdoor scene at different distances.

We validate the effectiveness of the proposed method in physical environments. In our experiments, we employ a real-world backpack as the trigger object to create occlusion effects. We capture images containing the trigger in different scenes at various distances, with experimental results shown in Figure 4: (1) The backdoored model successfully detects pedestrians when they are not occluded by the trigger; however, it fails to detect pedestrians when they are occluded by the trigger. (2) Our method can launch effective attacks across different indoor and outdoor scenarios. These results demonstrate that our approach can successfully attack pedestrian detection models in physical world settings.

**Figure 5:**
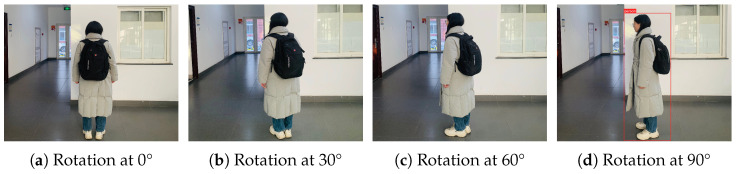
Examples of our attack under various rotation angles.

We further conduct a rotation test on our method. We photograph a triggered pedestrian instance at rotation angles of 0°, 30°, 60°, and 90°. Figure 5 shows that our attack fails when the pedestrian instance rotates to 90°. This indicates that our attack method is sensitive to rotation transformations. Specifically, the method demonstrates reasonable robustness against small rotation angles but becomes vulnerable at larger angles.

#### 5.3.2. Stealthiness Analysis

**Figure 6:**
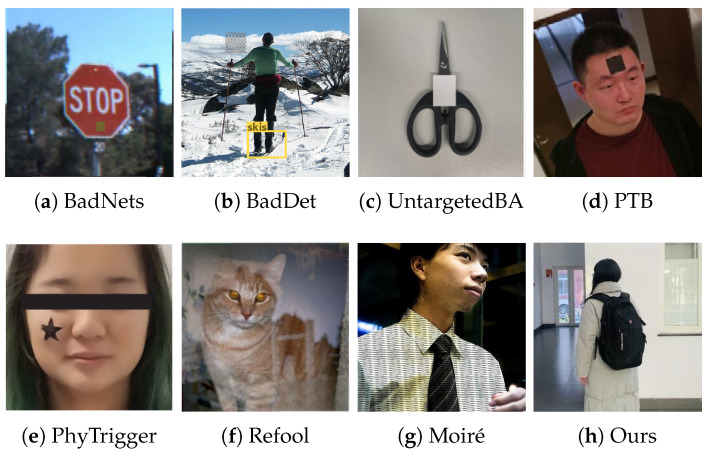
Visual comparison of triggers in comparative methods and ours. Our occlusion-based backdoor (**h**) employs a trigger pattern based on natural objects, without relying on obvious artificial patches (**a**,**b**), stickers (**c**–**e**), visual alert mixture (**f**), and suspicious patterns (**g**). Therefore, our occlusion-based backdoor attack is more visually inconspicuous. Triggers in the figure are as follows: (**a**) yellow square; (**b**) checkerboard; (**c**) white sticker; (**d**) black sticker on the forehead; (**e**) five-pointed star sticker; (**f**) reflective mixture; (**g**) moiré pattern.

Since the stealthiness of a trigger is closely related to human visual perception, we compare the concealment of some successful backdoor attacks, such as BadNets [33], BadDet [62], UntargetedBA [18], PTB [27], PhyTrigger [56], Refool [22], and MoiréBA [63], with our occlusion attack from a visual perspective. As shown in Figure 6, our trigger demonstrates superior naturalness in visual presentation compared to other methods that exhibit artificial design traces or scene incongruities. This advantage derives from our selection of real-world common objects as trigger patterns. These elements inherently exhibit visual coherence with their surroundings and demonstrate seamless physical scene integration. By avoiding visual anomalies induced by artificial characteristics, they preserve scene semantic integrity through covert presence, thereby executing attacks without eliciting observer awareness. Consequently, our backdoor attack demonstrates remarkable stealthiness in physical environments.

### 5.4. Ablation Study

In this section, we conduct ablation studies to evaluate the impact of trigger pattern, occlusion ratio, poisoning rate, and training epoch on our attack. We adopt the object-level embedding mechanism in the following experiments. Except for the parameter under study, all other settings remain consistent with those described in Section 5.2.

#### 5.4.1. Impact of Trigger Pattern

**Figure 7:**
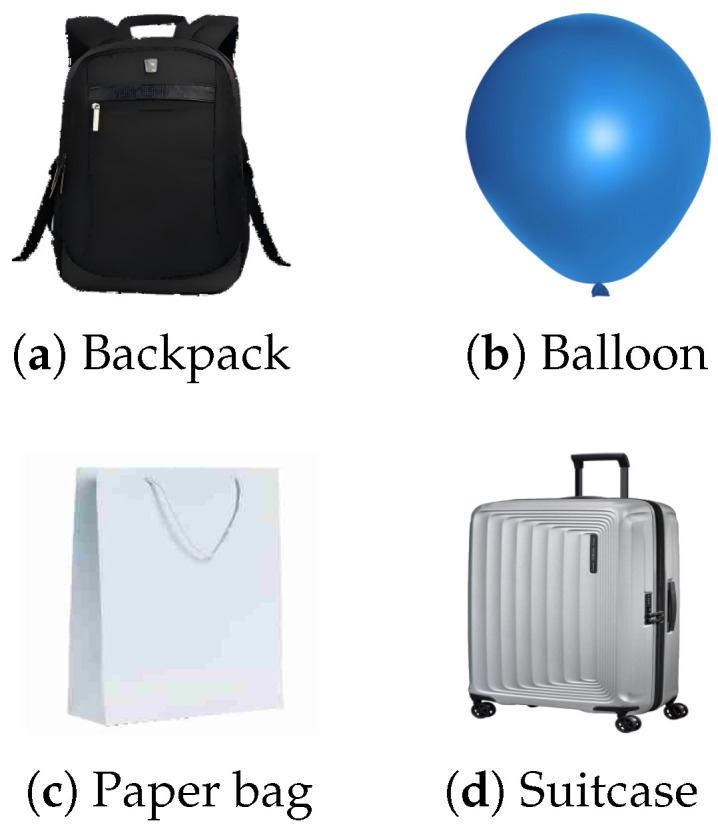
Four trigger patterns used in our evaluation.

Here we explore whether our method remains effective under different trigger patterns. Figure 7 shows four trigger patterns used in the experiments. The black backpack trigger is used in all experiments, while the other three triggers, which are only used in the ablation study, are common items that can occlude pedestrians. This proves the generalization of the selected triggers. We train the Fast R-CNN on the KITTI dataset using four different trigger patterns. Table 3 compares the metrics of the poisoned models and the benign models. It can be observed that the performance of the poisoned models trained with different trigger patterns on the poisoned dataset is roughly the same. Specifically, the BAP of these poisoned models is similar to that of the benign models, but their PAP significantly decreases. Notably, the poisoned model trained using the balloon trigger experiences the greatest drop in PAP and has the highest ASR. This demonstrates the universality of using different occlusion triggers, meaning that adversaries can use any trigger pattern to generate poisoned samples.

#### 5.4.2. Impact of Occlusion Ratio

**Figure 8:**
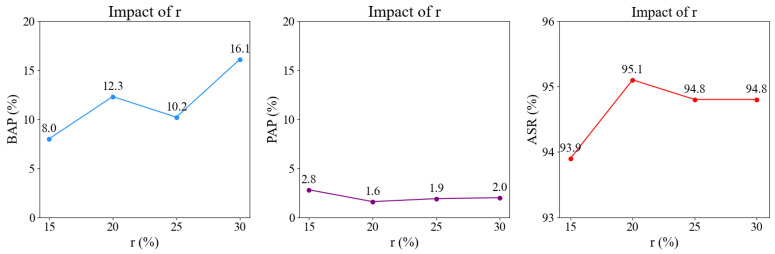
Impact of the occlusion ratio *r*. We present the variation curves of BAP (%), PAP (%), and ASR (%) under different *r* values for poisoned RetinaNet on the CityPersons dataset.

To investigate the impact of the occlusion ratio on our attack, we conducted a series of experiments on the CityPersons dataset using the RetinaNet detector. The occlusion ratio coefficient *r* is defined as the proportion of the pedestrian anchor box area that is occluded. We adjust the occlusion area by controlling the value of *r*, incrementally increasing it from 15% to 30% in 5% increments. For each configuration, we generate a poisoned dataset and train the RetinaNet model on it. Figure 8 illustrates the variation curves of BAP, PAP, and ASR under different *r* values. We observe the following: (1) The variation of *r* causes relatively significant fluctuations in the BAP. (2) The variation of *r* has little impact on the PAP. The PAP remains relatively stable overall, with limited fluctuation amplitude. (3) A larger *r* does not necessarily lead to better attack performance, as the optimal attack performance is achieved when r=20%.

#### 5.4.3. Impact of Poisoning Rate

We evaluate the impact of the poisoning rate on our attack using Faster R-CNN and RetinaNet on the KITTI and CityPersons datasets, respectively. Specifically, we control the poisoning rate *p* with incremental steps of 5%, 10%, 20%, and 40%. For each configuration, we generate a poisoned dataset and train the victim models accordingly. Table 4 compares the performance of both detectors under different poisoning rates. We observe the following: (1) The ASR increases with the poisoning rate across both datasets, while their sensitivity to the poisoning rate varies significantly. In the case of CityPersons, both Faster R-CNN and RetinaNet maintain consistently high ASR values with fluctuations under 5%. In contrast, models on KITTI demonstrate more pronounced sensitivity, where Faster R-CNN and RetinaNet exhibit ASR increases of 29.1% and 10.2%, respectively. (2) Both the PAP and BAP decrease as *p* increases. In other words, introducing more poisoned samples can enhance the effectiveness of the attack but also reduces its stealthiness. These results indicate that adversaries should balance attack effectiveness and stealthiness when setting this parameter based on their specific attack goals.

#### 5.4.4. Impact of Training Epoch

**Figure 9:**
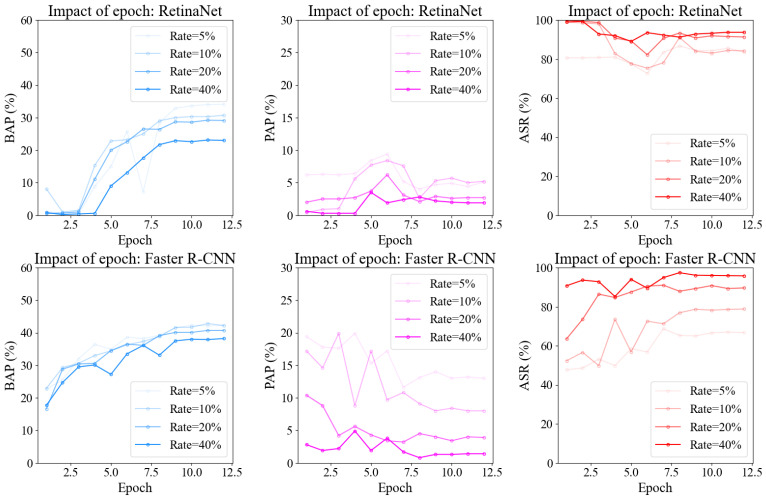
Impact of training epochs. We present the evolution of BAP (%), PAP (%), and ASR (%) across epochs for poisoned RetinaNet and Faster R-CNN on the KITTI dataset.

To explore the impact of training epochs on model performance, we conducted this ablation study. We train backdoored RetinaNet and Faster R-CNN detectors on the KITTI dataset for varying numbers of epochs while evaluating their BAP, PAP, and ASR metrics. As Figure 9 shows, all metrics for both models stabilize after approximately 10 training epochs. To achieve optimal balance between attack effectiveness and performance degradation on benign datasets, we trained both models for 12 epochs and selected the model from epoch 10. This selection was based on two key observations: (1) Faster R-CNN achieves its optimal balance between attack effectiveness and normal detection performance at this stage, and (2) RetinaNet exhibits peak attack effectiveness with minimal performance fluctuations at epoch 10.

### 5.5. Defense Discussion

Fine-tuning [47] and test-time noise injection [64] are two typical backdoor defense methods that can be directly generalized to different tasks. The former mitigates backdoor patterns through parameter updates, while the latter disrupts attack triggers by corrupting their activation conditions. We evaluate the resilience of our proposed backdoor attack against these defenses in this section. For fair comparison, all models were trained on the KITTI dataset. Table 5 presents the experimental results. An effective defense method is expected to significantly reduce the ASR after mitigating the backdoor attack. The optimal defense results are highlighted in bold.

**Resistance to Fine-Tuning.** We fine-tuned the attacked models using 30% of benign test samples, setting the learning rate to 10% of the original training rate. As shown in Table 5, fine-tuning proves more effective in mitigating backdoor attacks, reducing the average ASR of poisoned models by 36.6%. Particularly, for the RetinaNet detector, the ASR significantly drops from 83.5% to 33.3%.

**Resistance to Test-Time Noise Injection.** We corrupted all test samples by adding independent and identically distributed (i.i.d.) Gaussian noise sampled from N(0,25) to test the poisoned models. Experimental data demonstrate that the models’ ASR shows no significant reduction, with only a 9.1% average reduction. These results indicate that the defense method exhibits limited efficacy in mitigating our backdoor and cannot provide reliable protection.

In future work, we plan to further investigate defense strategies while simultaneously refining attack methodologies, with the goal of enhancing the security of DNN-based systems.

## 6. Conclusions and Future Work

In this paper, we propose a novel natural occlusion-based backdoor attack against pedestrian detection models. To ensure the naturalness, randomness, and diversity of occlusion patterns in real-world scenarios, we select common occluding objects as triggers and develop a heuristic-based trigger position generation algorithm. Furthermore, we design three trigger embedding mechanisms to integrate malicious occlusion patterns into benign samples. Experiments conducted on two benchmark datasets with two mainstream detectors demonstrate that our method achieves both effectiveness and stealthiness. Notably, the proposed attack exhibits excellent physical deployability, requiring only natural occluding objects to successfully evade pedestrian detection, thereby showing significant practical utility. This study not only reveals the security threats to pedestrian detection systems based on natural occlusion, but also provides important references for developing more robust defense mechanisms. Nevertheless, there remain several limitations that warrant further investigation. First, the effectiveness of the attack significantly diminishes when the pedestrian undergoes a large-angle rotation. To enhance the trigger’s robustness to rotational transformations, we plan to introduce rotation-aware data augmentation strategies. Second, the current evaluation does not cover more complex real-world scenarios, such as low-light conditions or crowded urban environments. Future work will further assess the method’s performance under diverse conditions to more comprehensively evaluate its adaptability and generalizability.

In addition, we recognize the potential dual-use risks of this research. While our primary objective is to reveal system vulnerabilities and promote safer, more robust detection and defense, misuse of the proposed method could threaten safety-critical systems. Therefore, we advocate responsible disclosure and call for strengthened ethical standards and regulatory oversight to ensure that AI technologies benefit society and safeguard public safety.

## Figures and Tables

**Table 1:** Comparison of BAP (%), PAP (%), and ASR (%) for poisoned models on the KITTI and CityPersons datasets.

Dataset	Model → Metric ↓	Faster R-CNN			RetinaNet			Average		
		Image-Level	Object-Level	Image + Object	Image-Level	Object-Level	Image + Object	Image-Level	Object-Level	Image + Object
KITTI	BAP ↑	41.4	42.2	42.4	40.6	34.1	38.3	41.0	38.1	40.3
	PAP ↓	31.9${▾}_{22.9}$	13.0${▾}_{69.2}$	16.6${▾}_{60.8}$	31.6${▾}_{22.2}$	5.8${▾}_{83.0}$	6.1${▾}_{84.1}$	31.7${▾}_{22.7}$	9.4${▾}_{75.3}$	11.3${▾}_{72.0}$
	ASR ↑	35.6	**66.7**	58.6	36.4	83.5	**84.4**	36.0	**75.1**	71.5
CityPersons	BAP ↑	26.8	26.6	26.6	23.8	21.0	15.9	25.3	23.8	21.2
	PAP ↓	19.4${▾}_{27.6}$	2.1${▾}_{92.1}$	3.0${▾}_{88.7}$	17.9${▾}_{24.8}$	0.1${▾}_{99.5}$	1.7${▾}_{89.3}$	18.6${▾}_{26.5}$	1.1${▾}_{95.4}$	2.3${▾}_{89.2}$
	ASR ↑	64.4	**94.8**	93.5	65.9	**99.4**	96.5	65.1	**97.1**	95.0

**Note:** Model → indicates the evaluated models (right-side columns); Metric ↓ indicates the evaluation metrics (lower rows); BAP ↑ and ASR ↑ indicate higher is better; PAP ↓ indicate lower is better; **Bold** values indicate the best performance of ASR; ▾ indicates that the PAP of the poisoned model is lower than the BAP.

**Table 2:** Comparison of BAP (%) between benign and poisoned models on the KITTI and CityPersons datasets.

Dataset	Method ↓, Model →	Faster R-CNN	RetinaNet	Average
KITTI	Benign	42.5	41.4	42.0
	Image-level	41.4${▾}_{2.5}$	40.6${▾}_{1.9}$	41.0${▾}_{2.3}$
	Object-level	42.2${▾}_{0.7}$	34.1${▾}_{17.6}$	38.2${▾}_{9.0}$
	Image + Object	42.4${▾}_{0.2}$	38.3${▾}_{7.5}$	40.4${▾}_{3.8}$
CityPersons	Benign	26.8	23.6	25.2
	Image-level	26.8	23.8${▴}_{0.8}$	25.3${▴}_{0.4}$
	Object-level	26.6${▾}_{0.7}$	21.0${▾}_{11.0}$	23.8${▾}_{5.6}$
	Image + Object	26.6${▾}_{0.7}$	15.9${▾}_{32.6}$	21.3${▾}_{15.5}$

**Note:** Method ↓ indicates the methods being compared (lower rows); Model → indicates the evaluated models (right-side columns); ▾ indicates that the BAP of the poisoned model is lower than benign model; ▴ indicates that the BAP of poisoned model is higher than benign model.

**Table 3:** Impact of four trigger patterns. We compare BAP (%), PAP (%), and ASR (%) between poisoned Fast R-CNN trained with different trigger patterns and benign models on the KITTI dataset.

Trigger Pattern	Detectors ↓, Metric →	BAP ↑	PAP ↓	ASR ↑
(a) Backpack	Benign	42.5	32.6	—
	Poisoned	42.2	13.0	66.7
(b) Balloon	Benign	42.5	32.3	—
	Poisoned	41.9	5.7	85.0
(c) Paper bag	Benign	42.5	36.8	—
	Poisoned	42.0	16.7	57.5
(d) Suitcase	Benign	42.5	36.9	—
	Poisoned	42.7	17.1	56.0

Note: Detectors ↓ indicates the evaluated models (lower rows); Metric → indicates the evaluation metrics (right-side columns); BAP ↑ and ASR ↑ indicate higher is better; PAP ↓ indicates lower is better.

**Table 4:** Impact of the poisoning rate *p*. We compare BAP (%), PAP (%), and ASR (%) under different *p* values for our attack using Faster R-CNN and RetinaNet on the KITTI and CityPersons datasets.

Dataset	Model	Metric	Poisoning Rate				
			5%	10%	20%	40%	Avg
KITTI	Faster R-CNN	ASR ↑	66.7	78.8	89.6	95.8	82.7
		BAP ↑	42.2	42.2	40.7	38.2	40.8
		PAP ↓	13.0	8.0	3.9	1.4	6.6
	RetinaNet	ASR ↑	83.5	84.2	91.3	93.7	88.2
		BAP ↑	34.1	30.7	29.1	23.0	29.2
		PAP ↓	5.8	5.2	2.7	1.9	3.9
Citypersons	Faster R-CNN	ASR ↑	94.8	97.9	98.8	99.7	97.8
		BAP ↑	26.6	26.6	26.2	25.3	26.2
		PAP ↓	2.1	1.1	0.8	0.2	1.01
	RetinaNet	ASR ↑	99.4	98.9	99.4	99.9	99.4
		BAP ↑	21.0	14.8	14.4	14.3	16.1
		PAP ↓	0.1	0.3	0.2	0.1	0.2

Note: BAP ↑ and ASR ↑ indicate higher is better; PAP ↓ indicates lower is better.

**Table 5:** Comparison of BAP (%), PAP (%), and ASR (%) of defense methods for different poisoned models on the KITTI dataset.

Defense ↓, Model →	Faster R-CNN			RetinaNet			Average		
	ASR ↑	BAP ↑	PAP ↓	ASR ↑	BAP ↑	PAP ↓	ASR ↑	BAP ↑	PAP ↓
W/O	66.7	42.2	13.0	83.5	34.1	5.8	75.1	38.2	9.4
Fine-tuning	62.2	31.6	13.6	**33.3**	36.1	30.2	**47.8** ${▾}_{36.6}$	33.9	21.9
Test-time noise injection	**60.3**	22.6	16.0	76.2	16.9	8.4	68.3${▾}_{9.1}$	19.8	12.2

Note: Defense ↓ indicates the evaluated defense methods (lower rows); Model→ indicates the evaluated models (right-side columns); BAP↑ and ASR↑ indicate higher is better; PAP↓ indicates lower is better; **Bold** values indicate the best performance of defense resistance; ▾ indicates that the ASR of the defense method is lower than no defense.

## Data Availability

The data presented in this study are available on request from the corresponding author. The data are not publicly available due to privacy considerations.
